# Supplementary material for: NCodR: A multi-class support vector machine classification to distinguish non-coding RNAs in Viridiplantae
Source: Quant Plant Biol. 2022 Oct 7;3:e23. doi: 10.1017/qpb.2022.18 (PMC10095871; doi:10.1017/qpb.2022.18)
Supplement: Supplementary file 1 [file S2632882822000182sup001.docx]

**Supplementary data for**

NCodR: A multi-class SVM classification to distinguish non-coding RNAs in Viridiplantae

Chandran Nithin^1,2+^, Sunandan Mukherjee^1,3+^, Jolly Basak^4^ and Ranjit Prasad Bahadur^1,^*

^1^Computational Structural Biology Lab, Department of Biotechnology, Indian Institute of Technology Kharagpur, 721302, India

^2^Laboratory of Computational Biology, Faculty of Chemistry, Biological and Chemical Research Centre, University of Warsaw, Warsaw 02-089, Poland

^3^Laboratory of Bioinformatics and Protein Engineering, International Institute of Molecular and Cell Biology in Warsaw, ul. Ks. Trojdena 4, PL-02-109 Warsaw, Poland

^4^Department of Biotechnology, Visva-Bharati, Santiniketan, 731235, India

^+^These authors have contributed equally and should be considered joint first authors.

*Corresponding author

Corresponding author: Ranjit Prasad Bahadur

# **Figure S1**


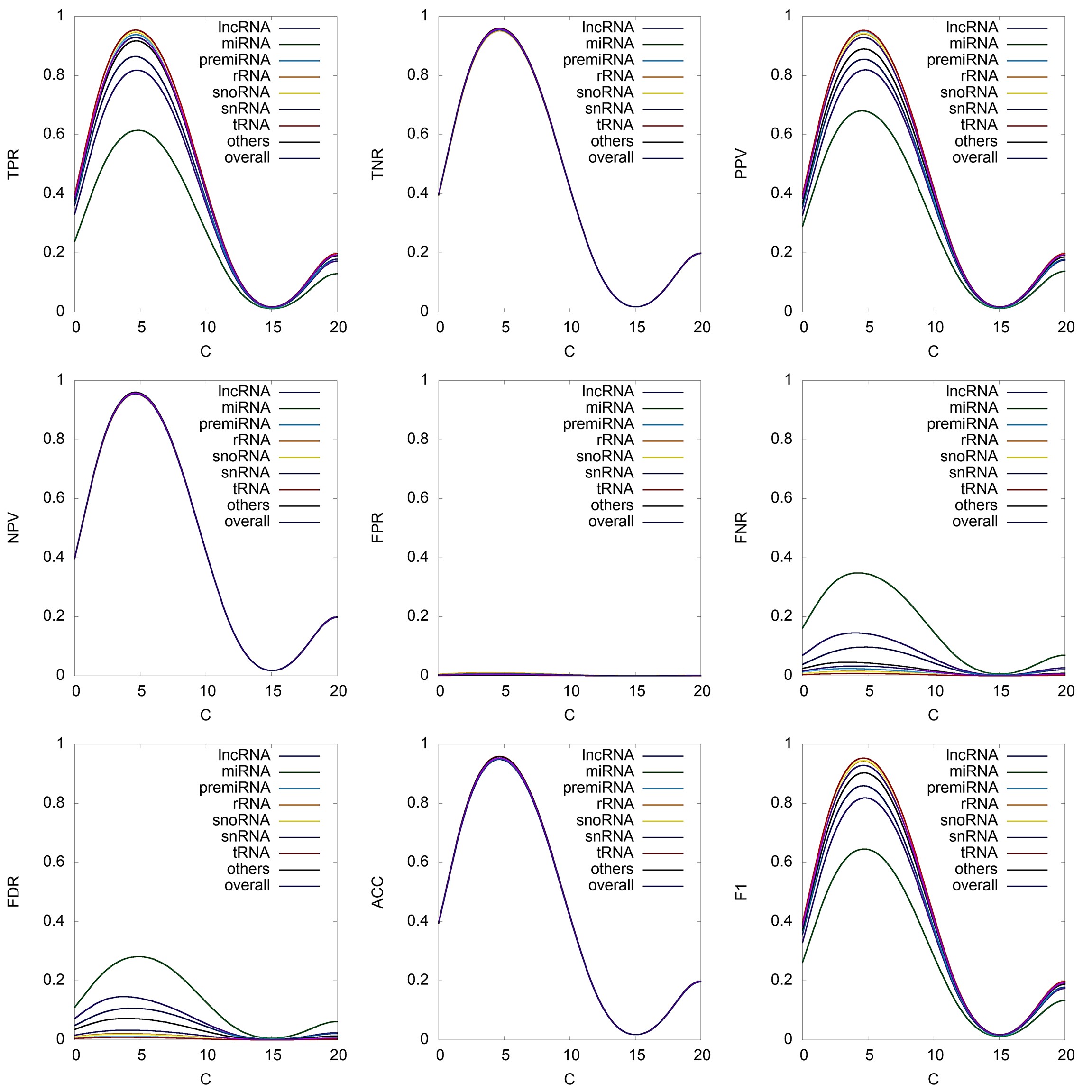


Figure S1: Performance measures of the SVM-RBF classifier for different values of C. For a constant value of hyperparameter gamma (gamma=0.003), the SVM-RBF classifier was trained for different values of C. The curves were smoothened using Gaussian kernel density estimates in the gnuplot program.

# **Figure S2**


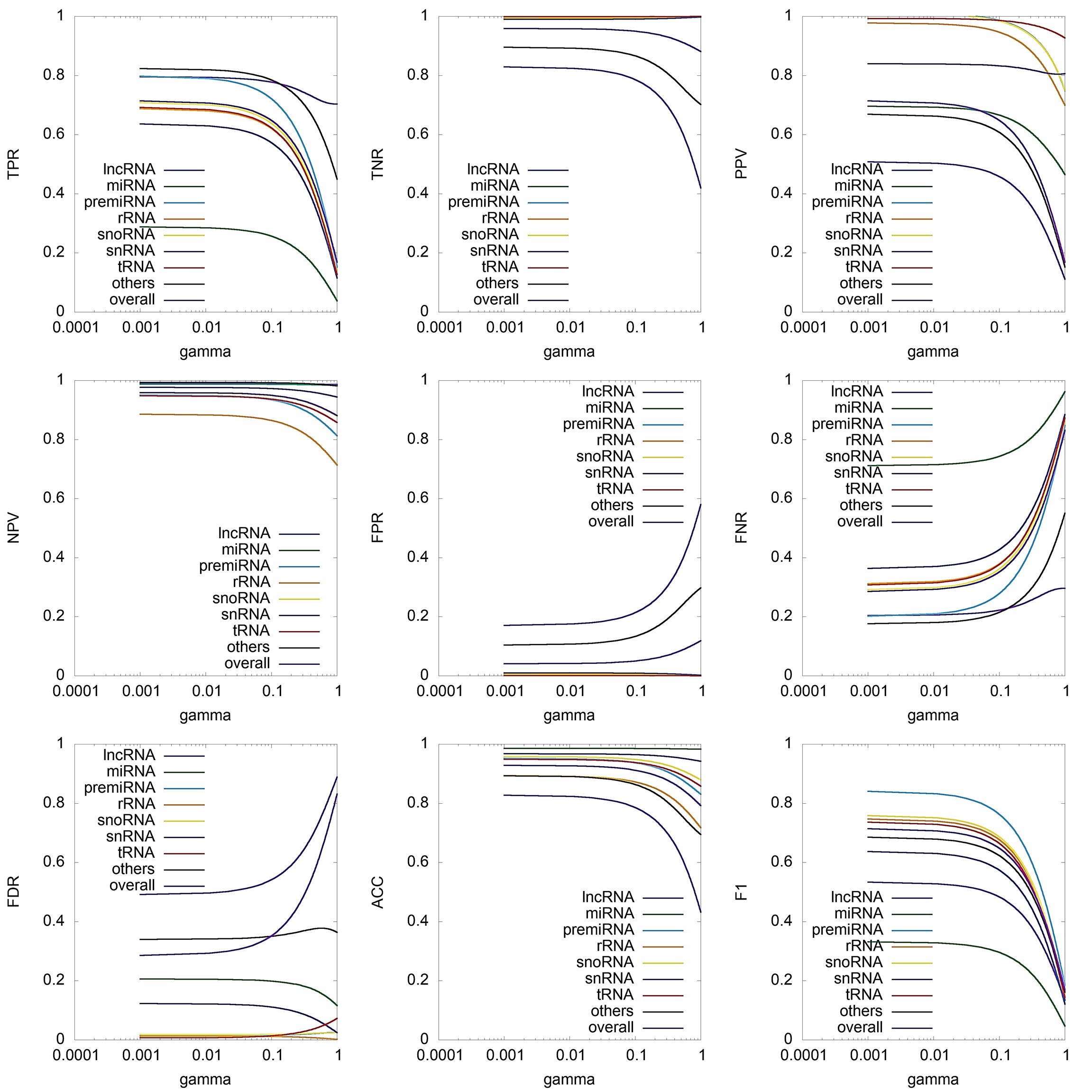


Figure S2: Performance measures of the SVM-RBF classifier for different values of gamma. For a constant value of hyperparameter C (C=8), the SVM-RBF classifier was trained for different gamma values. The curves were smoothened using *acspline* interpolation in the gnuplot program.
